# Assessing impacts of human-elephant conflict on human wellbeing: An empirical analysis of communities living with elephants around Maasai Mara National Reserve in Kenya

**DOI:** 10.1371/journal.pone.0239545

**Published:** 2020-09-18

**Authors:** Tobias Ochieng Nyumba, Olobeli Engini Emenye, Nigel Leader-Williams

**Affiliations:** 1 African Conservation Centre, Nairobi, Kenya; 2 Institute for Climate Change and Adaptation, University of Nairobi, Nairobi, Kenya; 3 Department of Geography, University of Cambridge, Cambridge, United Kingdom; 4 Kenya Agricultural and Livestock Research Organisation, Nairobi, Kenya; Feroze Gandhi Degree College, INDIA

## Abstract

Human-elephant conflict is an often intractable problem that threatens the contribution of conservation interventions to human wellbeing and securing livelihoods in Africa and Asia. Local human populations living in key elephant ranges are among the world’s most poor and vulnerable people. In efforts to address this problem, previous studies have mainly focused on the direct impacts of conflict and applied standard regression models based on the assumption of individual-level homogeneity. More recently, human-elephant conflict has been seen to extend well beyond the physical, to the psychological and social sides of wellbeing. However, the impacts on human wellbeing have not been robustly explored, especially for local communities co-existing with elephants. We evaluated the impacts of conflicts on the wellbeing of local communities around the world-famous Masai Mara National Reserve in Kenya. We conducted 18 focus group discussions with 120 community members in different locations and administered a questionnaire survey to 367 sampled households from 26 sub-locations in Trans Mara. We used descriptive statistics with appropriate statistical tests, including propensity score matching, to evaluate the impacts of conflict on human wellbeing. Before matching, the results of the descriptive statistics showed differences between households experiencing conflicts and those without in terms of gender, age, education level, household size, benefiting from elephant conservation, main occupation and number of income sources. Our matching results indicate the existence of a significant negative and positive impacts on four and one of our eight wellbeing indicators for households that experienced conflicts, respectively. Better conflict mitigation approaches and conservation policies need to be adopted to realize the harmonious and concurrent development of ecological and wellbeing objectives.

## Introduction

In the global south, the struggle to alleviate poverty often entails finding a balance between use and preservation of natural resources [1, 2]. Under these circumstances, conservation has not only been portrayed as both a win-win solution for poverty alleviation and sustainable development, but also as a constraint on economic growth [3]. In particular, conservation actions are expected to benefit human wellbeing, help secure livelihoods, yet pose little risk to the poor [2, 4, 5]. These benefits extend beyond human needs to how people use the resources to build their lives and achieve their notions of what it means to live well in their particular ecological context of wellbeing [6]. Consequently, the need to understand local people’s aspirations for their wellbeing has become central to the success of conservation interventions at the local level [6, 7].

In Africa, changes in human populations and land-use patterns have led to an increasing overlap of critical areas of conservation importance, and widespread exclusion of large mammal species from their previous ranges [8, 9]. Coincidentally, these large mammals are also important conservation flagship species [10–13], whose role in conservation goes beyond other conservation surrogates like keystone or umbrella species that are selected for their ecological functions only [14]. This is particularly true for African and Asian elephants (*Loxodonta africana*) and (*Elephas maximus*), respectively. Both species are commonly linked to some of the most intractable forms of conflict with rural communities [12, 14]. Human-elephant conflict (HEC) is not a new phenomenon in Africa [9, 15–18] and has evolved from being perceived as a nuisance to a major conservation concern across many elephant ranges [19, 20]. HEC is defined as “*any interactions between human and elephants which result in negative effects on human social*, *economic or cultural life; elephant conservation or on the environment*” [21]. According to Madden [22], HEC can be considered as a phenomenon where elephants negatively impact on human wellbeing or where the actions of people are detrimental to the survival of elephants.

HEC follows diverse impact pathways on human wellbeing [21, 23–27]. These include direct threats to life and loss of productive assets such as livestock [12] and subsistence crises where extreme crop-raiding is prevalent [28]. Furthermore, indirect threats caused by elephants can also impose curfews on school children and local residents through being closer to roads leading to schools and surrounding forests, further hindering access to essential social, cultural and economic services [29–31]. The impacts of other factors such as employment [32–34], income [35] and demographic variables [33, 36] have been highlighted in the literature. Furthermore, the interaction of these factors and underlying social, cultural, economic and conservation drivers are thought to contribute to complex and interdependent relationships between people and nature [37]. Such interactions may impact on the local support for conservation and development projects based on the flow of positive benefits or negative social impacts on people’s wellbeing [38, 39]. Focusing on wellbeing offers a platform for evaluating the broader spectrum of gains and losses due to conservation interventions. In turn, this should ensure fair and just distribution of conservation benefits, and mitigation of conservation-related costs on local people [1, 37, 40, 41].

HEC represents one of the most intractable problems of conservation affecting human wellbeing in Africa and Asia. This distinction is epitomised in key elephant ranges where the local human populations comprise some of the world’s poorest and most vulnerable regarding food security, health, education, infrastructure and social institutions [8, 42]. In Kenya, HEC is widespread in all wildlife ranges across the country [43]. However, key elephant ranges such as Trans Mara District (TM) along the south-western region are considered among the worst of HEC hotspots. Here both resident and migrating elephant populations from the adjacent Masai Mara National Reserve (MMNR) move into communal areas that are characterised by a mosaic of agriculture and elephant conservation activities [29, 44–46].

Human-elephant conflict has been documented in Kenya since the early 1990’s [e.g. 22, 42, 46–48]. These studies provide an invaluable foundation for the present research. However, their current limitations require further considerations for several reasons. Firstly, most previous studies have focused on direct measurable economic and material impacts of HEC, yet HEC is increasingly encompassing indirect, social, hidden and psychological sides of wellbeing [12, 29, 30, 49–52] and is highly differentiated [7, 29, 30, 50, 53, 54]. Furthermore, wellbeing is a multi-dimensional concept encompassing social, political, cultural, institutional and environmental dimensions [55, 56]. However, these studies tend to apply data aggregation approaches to assess mean impacts of HEC. In turn, this can mask inequalities [57], which nevertheless can be addressed by accounting for the differential impacts of HEC on human wellbeing [7].

Secondly, most previous studies have used ordinary regression models, which are assume homogeneity at the individual-level. However, the distribution of HEC is non-random, and this raises the problem of sample selection bias [58]. These studies have compared the impacts of HEC on households that experience HEC to those that do not experience HEC. This approach could lead to selection bias, leading to overestimating or underestimating impacts of HEC. Therefore, a meaningful way of measuring the impacts of HEC would be to compare impacts on the same households with and without HEC as a means to addressing the selection bias. This would require different approaches to simulate the random experimental process and to estimate the treatment effect, based on the condition that the treatment group and control group are as similar as possible.

The objective of this study was to assess the impacts of HEC on the wellbeing of local residents in TM. Specifically, the study sought to examine three questions:

- What factors affect human wellbeing?
- What is the effect of the HEC on the wellbeing of local residents in TM?
- Compared to the control group, what is the effect of the HEC on the wellbeing of households experiencing HEC?

## Materials and methods

This study was approved by the Ethics Review Group of the University of Cambridge, and the protocols used in the study were approved by the National Council for Science and Technology and Innovation of the republic of Kenya (Permit No. NACOSTI/P/14/0362/2798) and the Kenya wildlife Service (Permit No. KWS/BRM/5001). A total of 367 local residents were interviewed between 2014 and 2015 in the Mara Ecosystem, Kenya and informed consent was sought according to the University of Cambridge Research Ethics guidelines and strategies aimed at minimizing harm to the subject.

### Description of the study area

Trans Mara (TM) District lies in the south-west of Kenya on the border with Tanzania (0°50′−1°50′S, 34°35′− 35°14′E). The district covers an area of some 2900 km^2^ and encompasses the western portion of the world-famous Masai Mara National Reserve (MMNR) (S1 Fig). Approximately 2200 km^2^ of TM is unprotected and is occupied by local communities separated from the protected MMNR by the steep Oloololo escarpment. Land use in TM comprises cultivation, livestock keeping, forests, rural and urban settlement, mining, and wildlife conservation and tourism [45]. Wildlife-based tourism in the Mara ecosystem accounts for over 18% of the annual tourist visits to Kenya and is worth an estimated US$15–20 million [59]. A range of private and communal wildlife-based enterprises including campsites, tented camps, airstrips, balloon safaris and lodges exist within the communal area. These facilities contribute to the economic standing of TM through direct and indirect employment in the catering, administrative and tour operations functions, among others. In addition, local residents sell food products to the lodges, as well as Maasai cultural items such as embroidery and woodcarvings [60]. TM has a bimodal rainfall pattern from March to June and from November to December. The district experiences an annual average of between 1200–1500 mm of rainfall, with a north-south gradient of high to low rainfall across TM. The natural vegetation is a mosaic of Afro-montane, semi-deciduous and dry deciduous forests and Acacia savannah [61]. However, many areas of TM have high agricultural potential and cultivation is widespread [62]. The remaining forest provides refuge for a resident, unprotected population of 200–300 elephants that once ranged across most parts of TM district, but that now extend over 1,000 km^2^ [29, 63].

The human population of TM has witnessed a rapid increase estimated at 274,532 during the 2019 national census with a population growth rate of 3.3% compared to 2.2% in national estimates. The average household size in TM is 5.03 which is higher than the national household size of 4.4 [64]. TM has seen a dramatic increase in agriculture, land sub-division and fencing of such parcels [44]. With a population of about 300 resident elephant population and over 3000 transient animals, HEC has been inevitable in TM [44]. The conflict between elephants and people over cultivated crops began with the immigration of non-Maasai into TM in the 1920s [65]. As cultivation was increasingly introduced to TM District by these immigrants, so too did crop-raiding increase to become a perennial problem throughout the 1990s and 2000s. Both people and elephants suffer injury and death, and attitudes towards elephants in TM District are generally negative [29, 45, 66]. Rural communities receive little support from the national wildlife authority, Kenya Wildlife Service, because of limited resources and personnel [44, 62].

From 2003, the World Wide Fund (WWF) for Nature’s Kenya programme in TM initiated a series of HEC mitigation strategies which included community awareness and education and testing a set of farm-based HEC mitigation tools [46]. These tools, including chilli-grease fences, tree-top watchtowers, thunderflashes, powerful torches, cowbells, fires and a standard wire fence erected on the elephant corridors, mainly sought to empower local communities to address HEC with little or no external support. Nyumba [29] established that these techniques increased the awareness of HEC mitigation approaches and created a legacy of improved attitudes towards elephants and capacity for HEC mitigation. Nevertheless, the overall uptake was limited. Since then various community based and research and conservation organisations such as the Mara Elephant project have continued to work with communities to expand the application of these techniques including aerial surveillance using helicopters to mitigate HEC and minimise illegal killing of elephants and other wildlife in the landscape. However, both the financial and non-financial costs of establishing and implementing HEC mitigation methods mean that they are often poorly or patchily implemented [29]. As a result, local people have little faith in these methods [29, 45, 62]. Therefore, TM represents a model of a situation common across Africa where elephants and people co-exist in disharmony.

### Data collection

We conducted focus group discussions to identify and define the components of wellbeing among local residents in TM following White and Pettit [67] and Cahyat *et al*. [68]. We used a combination of multi-stage and simple random sampling techniques to draw a sample of respondents [69] for our household interviews. Firstly, based on the specific administrative divisions falling within the elephant range in TM, and secondly by specific sub-locations where elephant presence was determined to be permanent, recent or erratic based on sightings during the past five years. Consequently, we focused on 26 sub-locations. Secondly, we clustered the households according to administrative boundaries. In this case, “sub-location” was assumed to be the unit of clustering. Finally, we drew a random sample of households from within each cluster based on proportional random sampling [70]. We selected a total of 376 households which were considered adequate within a margin of error of 5% and a 95% confidence level. Here, a “household” is defined as a person or group of persons related or not, residing in the same homestead or compound. However, all household members do not necessarily live in the same dwelling unit, but do share the same cooking arrangements, and are answerable to the same household head [71].

We recruited local assistants to assist in the administration of the questionnaires. Although the assistants had some prior research experience, they were further trained in the use of the questionnaires to address the specific needs of this study. The questionnaire survey was administered to household heads. However, in their absence, another adult (>18years) was interviewed. While this might affect the comparability of the data gained, it was justified as a pragmatic choice given the realities of the fieldwork context. In all, 367 questionnaires were completed, with a response rate of 97.6%. The household survey recorded household socio-demographic data; human, material and social resources; wellbeing assessment; and interaction with elephants. The short version of the questionnaires can be found in supplementary materials (S1 Appendix).

### Data analysis

To measure the impact of human-elephant conflict (HEC) on the wellbeing of rural households, we used the technique of Propensity Score Matching (PSM). This enabled us to extract from the sample of households that had experienced HEC a set of matching households that had not experienced HEC in all relevant pre-intervention characteristics, following Caliendo and Kopeinig [72] and Rosenbaum and Rubin [73]. Households that had experienced HEC were considered the “treatment sample” whereas those that had not experienced HEC were considered the “control sample” that were used for comparison. Meanwhile, the change in wellbeing scores was considered the “impact of HEC” and was the outcome indicator. We attempted to estimate the average impact of treatment on treated (ATT) following Caliendo and Kopeinig [72]. We were interested in two sets of variables: the outcome variable in this study referred to the wellbeing indicators and the choice of variables for estimating PSM.

#### Human wellbeing indicators

The first step towards measuring wellbeing was the construction and validation of wellbeing indicators. First, we conducted a qualitative analysis of the focus group discussion data to identify broadly the conception and understanding of factors that constitute wellbeing for the TM residents. We then used IBM SPSS Statistics 22 [74] software to conduct a series of statistical steps. These included the test for assumptions of normality and suitability of the data for factor analysis and factorial analytic techniques to reduce variables, validate and assign scores to the various wellbeing indicators. We generated 10 factors as depicted in (S2 Appendix). We tested the 10 factors for internal consistency using Cronbach’s alpha and inter-item correlations [75]. We evaluated the Cronbach’s alpha coefficients using the guidelines suggested by George and Mallery [76: 231] where >0.9: Excellent; >0.8: Good; >0.7: Acceptable; >0.6: Questionable; >0.5: Poor, and <0.5: Unacceptable. Our results in (S2 Appendix) show that the Cronbach’s alpha coefficients for our items ranged from 0.07 to 0.81. Therefore, we established that only eight out of the 10 factors met the criteria for inclusion having scores of >0.6. Two factors, Support Programmes Index and Health Index had Cronbach's alpha coefficient <0.5, indicating unacceptable reliability and were therefore dropped from subsequent analyses. Our final list of eight indicators included people‘s assessment of their wealth (Wealth Index, WI), education (Education Index, EI), food security (Food Security Index, FSI), satisfaction with services (Satisfaction with Services Index, SSI), social interaction (Social Index, SI), access to services (Access to Services Index (ASI) as well as perceptions of the state of the natural environment (Natural Sphere Index, NSI). To facilitate measurements, we developed score bins with: scores of 33.5 or less indicating a low wellbeing score; scores between 33.6 and 66.4 indicating a moderate wellbeing score; and scores of 66.5 or higher indicating a high wellbeing score.

#### Estimation of propensity scores and matching households

Propensity scores matches (PSM) can be obtained through standard probability models such as logit, probit or multi-nominal logit. We adopted a logit model to estimate PSM using a composite of pre-intervention characteristics of the sampled households [73]. Brookhart *et al*. [77] outlined three options for the selection of pre-intervention characteristics for the propensity model as follows: i) the probability of receiving a treatment (i.e., experiencing HEC); ii) the outcome variables (i.e., wellbeing); or iii) both the probability of treatment and the outcome. For this study, we applied option (ii), and included all pre-intervention characteristics in the PSM model that were significantly related to the outcome variable, that is, wellbeing scores. In estimating the logit model, we used “experiencing HEC” as the binary response which took the value of 1 if a household experienced HEC and 0 if otherwise. This resulted in a continuous variable where the probability of observing two units (i.e., households) with exactly the same propensity score is, in principle, zero.

We used the Propensity Score Matching tool in SPSS to calculate propensity scores and applied 1:1 matching with a caliper of 0.1 [78], to evaluate the matching using Rubin [79]’s criteria. We used the propensity scores to match households that had experienced HEC with those that had not experienced HEC, by imposing a common support condition to ensure that any combination of characteristics observed in the treatment group could also be observed among the control group, following Bryson and Richard [80] and Caliendo and Kopeinig [72]. Finally, we evaluated the matching quality using Standard bias, t-test, joint-significance and pseudo-R^2^ to check if our matching procedure was able to balance the distributions of the propensity scores [72] between households that had experienced HEC and those that had not. In determining the impact of HEC on human wellbeing, we ran a regression analysis with different wellbeing scores and HEC before and after matching. A negative value of ATT suggests that households who had experienced HEC had lower wellbeing scores than households that had not experienced HEC, and the reverse also held true.

## Results

### Descriptive statistics

Results of the descriptive statistics before matching showed differences between households experiencing conflict, and those without conflict, in terms of gender, age, education level, household size, benefiting from elephant conservation, main occupation, ethnicity and number of income sources (S1 Table). Furthermore, there were differences in wellbeing scores between households that had experienced HEC and those that had not experienced HEC. Those experiencing HEC reported poor scores on “Satisfaction with services” (49.16 vs 53.04), “Subjective wellbeing” (46.56 vs 54.59) and “Social interaction” (41.38 vs 51.75) before matching. However, after matching, HEC households reported a huge decline in “Asset ownership” (48.43 vs 58.70) but reported an improvement in “Food security” (49.87 vs 43.57) and “Access to services” (55.95 vs 47.08). Marginal improvement was observed on “Natural environment” (48.95 vs 48.81) (S2 Table and S2 and S3 Figs).

#### Estimation of the propensity scores

The logit estimate appeared to perform well for the intended matching exercise. The pseudo-R^2^ value of 0.223 shows that households did not have many distinct characteristics overall. Therefore, finding a good match between the treated and control households became easier. The maximum likelihood estimates of the logistic regression model result show that experiencing conflict with elephants was significantly influenced by eight variables (S1 Table). These include gender, education, age, ethnicity, occupation and income sources of the household head. In addition, size of the household and whether the household benefited from elephant conservation or not significantly influenced experiencing conflict with elephants in TM.

Furthermore, we assessed the distribution of PSMs for households that experienced HEC and those that did not, before imposing the common support condition, which involves comparing the minimum and maximum scores for each treatment group. The households in our study had mean PSM’s ranging from 0.5 to 0.7 for those that experienced HEC and those without HEC, respectively (S3 Table). The basic criterion for determining the common support is to delete all observations whose propensity score is smaller than the minimum of one group and larger than the maximum in the opposite group [72]. For our sample, the means of estimated PSMs for households that experienced HEC and those that did not, varied considerably (S3 Table). Therefore, our common support region would then lie between 0.1347 and 0.9325. As a result of this restriction, 109 households mainly from those that experienced HEC, were dropped from the analysis to estimate the average treatment effect. Propensity score means after matching were found to be 0.56 ±0.17 for households that had experienced HEC and 0.56 ± 0.17 for households that had not experienced HEC, respectively. These similar means indicate that the difference of the mean propensity scores of the two groups lay within the recommended limit of 0.5 standard deviations. The ratio of the propensity score variances in the two groups was 1, which is the optimal ratio as indicated by Rubin [79].

Finally, we examined the matching using an independent sample t-test. The estimate of the probability of a household experiencing HEC was significant (t(225.97) = 4.682, *p*≤ .005) before matching and showed no significant differences (*t*(214) = -.012, *p* = .991) between the households that experienced HEC and those that did not experience HEC on any of the pre-intervention characteristics included in the model, indicating that the new set of households had equal chances of experiencing HEC (S4 Table).

The t-values also indicated that at least six of the selected covariates showed statistically significant differences before matching. The process of matching all the covariates and the propensity scores, resulted in statistically insignificant differences. This implies, that our matching process successfully created a covariate balance between the treatment and control samples, thereby enabling us to proceed with the analysis. The matched sample subsequently included a total of 216 households, evenly distributed across the two groups, one that had experienced HEC and another that had not experienced HEC.

#### Factors affecting human wellbeing

We used generalised linear models (GLMs) in SPSS to explore a range of factors affecting various indicators of human wellbeing scores. For this study, continuous explanatory variables included age and household size, whereas categorical explanatory variables included gender, education level, employment, benefits form conservation and human-elephant conflict. The results of the analysis showed that households that did not experience HEC had a significantly higher probability of improving their scores of wealth (WI), satisfaction with services (SSI), education (EI) and social (SI) indicators of wellbeing. However, the same households had a significantly higher probability of gaining poor scores on access to services indicator (ASI). Other variables, in particular, age, household size, receiving benefits from conservation, and having no formal education influenced different indicators. Gender, employment and having formal (primary and secondary) level of education did not appear to influence any of the wellbeing indicators (S5 Table).

#### Impacts of HEC on human wellbeing

We applied generalised linear models (GLM) in SPSS to accommodate non-normal responses as well as a non-linear relationship between the expectation of the response and the covariates. We ran the regression in terms of main effects using wellbeing indicators; Subjective wellbeing, Wealth indicator, Access to services, Food security, Satisfaction with services, Education, Social interactions, and Natural environment, for both unmatched and matched data samples. S6 Table shows the results before matching which indicates that HEC only affected Subjective wellbeing and Social interactions. All the other indicators were not impacted by HEC. S7 Table shows the results for the matched household samples. Our matching results indicate the existence of a negative significant impact on four and positive impact on one of the eight wellbeing indicators for households that experienced HEC. However, the matching process showed that no significant difference existed in the scores of “Subjective wellbeing, Food security and Natural environment.

## Discussion and policy implications

Our study showed that households experiencing HEC reported low wellbeing scores on most dimensions compared to those without HEC. This finding is consistent with the assertion that HEC impacts do not always affect the community uniformly [50, 53, 54]. The measurement of Wealth indicator in this study was based on the diversity and regularity of income sources, and ownership of a motorcycle following Nyumba [29]. HEC, especially damage to crops and property, poses a threat to livelihood diversification activities and agricultural production. Previous research suggests that damage to crops and attacks on humans led to increased workloads and diminished physical and financial wellbeing, as well as by reducing capacity to take part in income generation activities [50, 53]. Furthermore, these households invest in crop, livestock and personal protection strategies, and repair of damaged property which draws from their personal income and savings. Consequently, they suffer opportunity costs linked to livestock herding, crop guarding and personal protection [12, 13].

Previous studies among communities living around PAs in Kenya established that government agencies imposed “official curfews” on social gatherings in response to frequent movement of wildlife through areas of human settlement [29, 45, 81]. In addition, elephants impose natural curfews, by restricting human movements, disrupting social and economic activities, and by their close presence and heavy use of communal lands adjacent to PAs. TM represents a landscape that is used by elephants as a dispersal area and hence experiences regular interactions with humans. Consequently, elephant presence imposes a restriction on local people and thereby contributes to the low scores in social interaction among the households experiencing HEC. Such restrictions and disruptions have also been reported among school-going children in TM [29, 30]. It is not surprising that HEC is reported to impact on the educational wellbeing in this landscape. Consequently, elephant presence compromises peoples’ wellbeing where relations with community and family members and quality education in TM are concerned.

Our study also highlighted two positive outcomes on access to services (ASI) for households experiencing HEC. Elephant conservation in TM has received much attention from the government, private investors, local communities and international conservation organisations through investments in nature-based tourism, infrastructure and conservation research [e.g. 43]. According to Poole *et al*. [44], elephants play a critical role in the Mara ecosystem from biodiversity, tourism and human development perspectives. Recent developments in TM have involved the rehabilitation of footpaths and dirt roads, thereby making movement between locations faster and easier [29]. Furthermore, motorcycle use has grown over the last few years in TM as an affordable means of transport, giving access to services in the community [29, 82]. Households that do not own motorcycles access them through paying transportation costs or hiring motorbikes from their neighbours to access the services. Although neighbouring locations without elephant conservation-related investment have also seen some development, mainly from the county government, the levels of investment have not matched those found in the elephant range in TM. For example, artisanal mining facilities are found in central Lolgorian and Keyian divisions, and largescale sugar cane production and processing in Kilgoris and Pirrar division. These facilities have come with some infrastructure improvement, but these are limited to the specific sites of mining and sugar processing areas. Therefore, their wider utilisation by the community to access essential services is very minimal.

Our results indicate that education, age, household size, employment and receipt of benefits from elephant conservation significantly affected wellbeing in TM. However, HEC appeared to have broader impacts than previously documented, affecting five out of the eight wellbeing indicators in TM. Food security was negatively impacted by age and household sizes. Since local people in TM draw their livelihoods from agro-pastoral activities, elderly individuals and larger households were prone to food insecurity as their productivity diminished with age and became stretched with large households. Locals in TM are largely agro-pastoralists, a livelihood activity that does not require any formal education to accomplish, since it is a traditional way of life in the landscape. One of the outcomes of education is better opportunities for formal employment and hence wealth creation. However, households in TM without formal education (S5 Table) had a marginal positive outlook on wealth indicator compared to those with higher educational achievement. Studies have shown that individuals in employment have increased economic power and can purchase goods and services from other community members, can interact outside their local communities and access more information [83]. In TM, formal employment was not readily available. However, most residents were engaged in manual and unskilled labour and businesses which afforded them opportunities to make money. However, the number of people in employment is very low, and they are employed in lower cadres of employment with lower wages. Therefore, income from employment does not seem influential in mediating wellbeing in TM. Indeed, most of the residents derived their financial power from the sale of livestock and grains, and taxi and motorbike operations.

In conclusion, our results suggest that HEC has serious implications for the wellbeing of local communities living with elephants. There is need to promote conservation activities that enhance wellbeing and mitigate the negative impacts of HEC. There is an urgent need to strengthen the capacity and the knowledge of the local people sharing their landscape with elephants to enable them cope with HEC. One way of doing this is to strengthen the traditional HEC mitigation techniques to complement the centralised, and often resources-starved mechanisms. Communities and conservation organisations are already testing different HEC mitigation techniques that confer control and responsibility to local communities, thereby offering real opportunities for collaboration in HEC mitigation. Secondly, we suggest that conservation and other relevant actors consider the promotion of livelihoods diversification as a route to improving the wellbeing of rural residents and biodiversity conversation. Studies have demonstrated that diversification reduces vulnerability and poverty, increases income and wealth, enhances security and can improve the quality and sustainability of the natural resources that constitute key assets in rural livelihoods, and hence wellbeing [84, 85]. This study has also identified from the unmatched samples that HEC had a significant impact on subjective wellbeing. However, this impact diminished for the matched samples. Other studies have identified psychological impacts of HEC [86–88]. Although we could not establish such in the present study, we do recommend more collaborative study to establish the existence and extent of such impacts in order to recommend more focused interventions such as psychological counselling.

## Supporting information

S1 Fig Trans Mara district. (TIF)

S2 Fig Wellbeing before matching. (TIF)

S3 Fig Wellbeing after matching. (TIF)

S1 Table Logit estimates. (DOCX)

S2 Table Wellbeing scores. (DOCX)

S3 Table Distribution of PS. (DOCX)

S4 Table PS and covariate balance. (DOCX)

S5 Table Factors affect WB. (DOCX)

S6 Table Impacts of HEC WB. (DOCX)

S7 Table Impacts of HEC WB. (DOCX)

S1 Appendix Household questionnaire. (DOCX)

S2 Appendix Wellbeing indicators development. (DOCX)

S3 Appendix Testing assumptions of linear regression. (DOCX)
