# Supplementary figures and images for: Assessing impacts of human-elephant conflict on human wellbeing: An empirical analysis of communities living with elephants around Maasai Mara National Reserve in Kenya
Source: PLoS One. 2020 Sep 18;15(9):e0239545. doi: 10.1371/journal.pone.0239545 (PMC7500588; doi:10.1371/journal.pone.0239545)

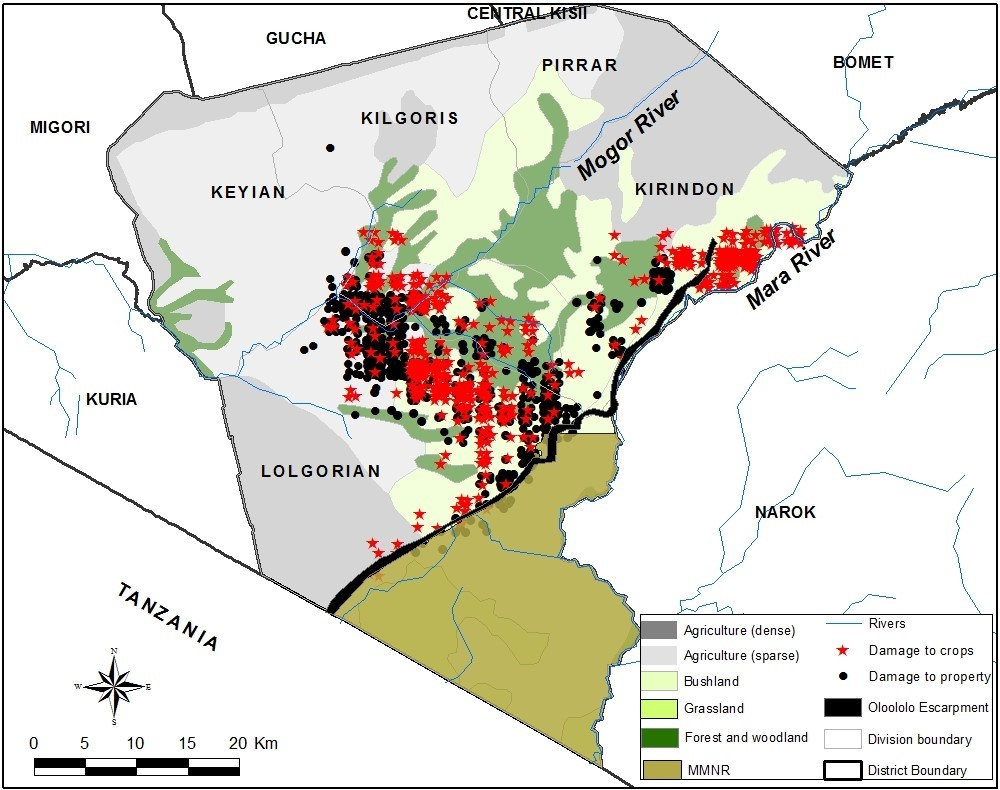

Supplement: S1 Fig — (TIF) [file pone.0239545.s001.tif]

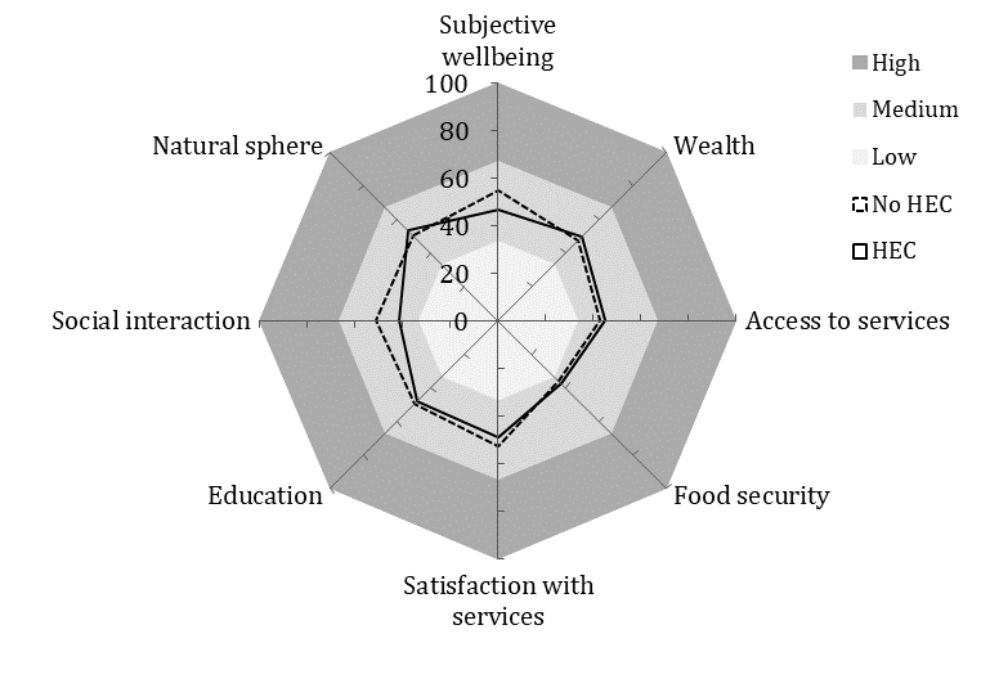

Supplement: S2 Fig — (TIF) [file pone.0239545.s002.tif]

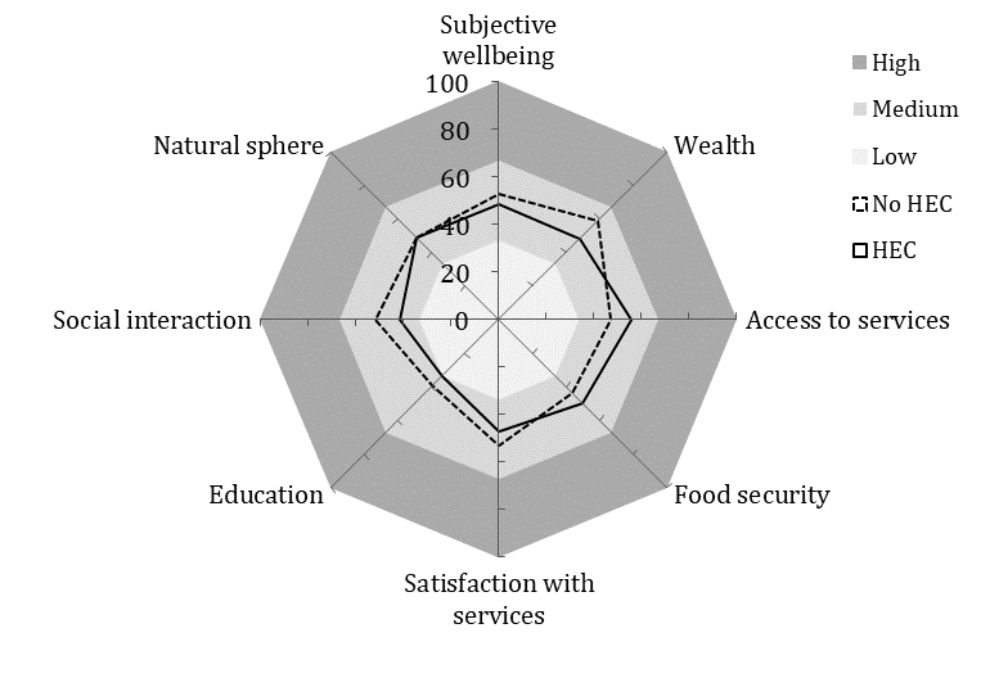

Supplement: S3 Fig — (TIF) [file pone.0239545.s003.tif]
