# Supplementary material for: Assessing impacts of human-elephant conflict on human wellbeing: An empirical analysis of communities living with elephants around Maasai Mara National Reserve in Kenya
Source: PLoS One. 2020 Sep 18;15(9):e0239545. doi: 10.1371/journal.pone.0239545 (PMC7500588; doi:10.1371/journal.pone.0239545)
Supplement: S1 Table — (DOCX) [file pone.0239545.s004.docx]

S2 Table: Logit estimates for the propensity scores (n=363)

| **Logit specification** | **Coef.** | **Std. Err** | **Z** |
| --- | --- | --- | --- |
| Gender | .688 | .287 | **5.759*** |
| Age of respondent | -.029 | .010 | **8.877**** |
| Education level of respondent | .310 | .141 | **4.840*** |
| Ethnicity of respondent | -1.021 | .287 | **12.702***** |
| Household size | .109 | .058 | **3.537**** |
| Benefits from elephant conservation | .651 | .242 | **7.245**** |
| Occupation of respondent | .250 | .088 | **8.131**** |
| Number of income sources | .636 | .198 | **10.358***** |
| Number of assets owned | .130 | .243 | .284 |
| Participation in decision making | -.257 | .163 | 2.487 |
| Constant | -1.574 | 1.016 | 2.399 |
| Log-likelihood | 430.041 |  |  |
| Pseudo R^2^ | .223 |  |  |
| Chi^2^ | 66.429 |  |  |
| *P* | .000 |  |  |

Dependent variable = 1 if the household experienced conflict with elephants and 0 if otherwise.

Asterisks *, ** and *** indicate statistical significance at the 10% and 5% and 1% levels respectively.
