# Supplementary material for: Assessing impacts of human-elephant conflict on human wellbeing: An empirical analysis of communities living with elephants around Maasai Mara National Reserve in Kenya
Source: PLoS One. 2020 Sep 18;15(9):e0239545. doi: 10.1371/journal.pone.0239545 (PMC7500588; doi:10.1371/journal.pone.0239545)
Supplement: S2 Table — (DOCX) [file pone.0239545.s005.docx]

**S1 Table: Wellbeing scores before and after matching of the households**

|  |  | **Unmatched Sample** | | **Matched Sample** | |
| --- | --- | --- | --- | --- | --- |
| **Wellbeing Indicator** | **Sample** | **Mean** | **Difference** | **Mean** | **Difference** |
| Subjective wellbeing | Treatment | 46.56 | -8.03 | 48.40 | -4.84 |
|  | Control | 54.59 |  | 53.24 |  |
| Asset ownership | Treatment | 50.12 | 2.66 | 48.43 | -10.27 |
|  | Control | 47.46 |  | 58.70 |  |
| Access to services | Treatment | 44.75 | 2.02 | 55.95 | 8.87 |
|  | Control | 42.74 |  | 47.08 |  |
| Food security | Treatment | 37.17 | 1.08 | 49.87 | 6.30 |
|  | Control | 36.09 |  | 43.57 |  |
| Satisfaction with services | Treatment | 49.16 | -3.88 | 47.01 | -6.03 |
|  | Control | 53.04 |  | 53.04 |  |
| Education | Treatment | 48.30 | -1.12 | 33.93 | -5.48 |
|  | Control | 49.41 |  | 39.41 |  |
| Social interaction | Treatment | 41.38 | -10.37 | 41.69 | -10.06 |
|  | Control | 51.75 |  | 51.75 |  |
| Natural environment | Treatment | 53.77 | 3.49 | 48.93 | 0.12 |
|  | Control | 50.28 |  | 48.81 |  |
