# Supplementary material for: Assessing impacts of human-elephant conflict on human wellbeing: An empirical analysis of communities living with elephants around Maasai Mara National Reserve in Kenya
Source: PLoS One. 2020 Sep 18;15(9):e0239545. doi: 10.1371/journal.pone.0239545 (PMC7500588; doi:10.1371/journal.pone.0239545)
Supplement: S3 Table — (DOCX) [file pone.0239545.s006.docx]

S3 Table: Distribution of estimated propensity scores

| **Groups** | **Number** | **Mean** | **Std. Error** | **Min** | **Max** |
| --- | --- | --- | --- | --- | --- |
| All households | 325 | .6284833 | .01005286 | .12825 | .94137 |
| Households with conflict | 217 | .6606680 | .01216052 | .12825 | .94137 |
| Households without conflict | 108 | .5638159 | .01620294 | .13468 | .93246 |
