# Supplementary material for: Assessing impacts of human-elephant conflict on human wellbeing: An empirical analysis of communities living with elephants around Maasai Mara National Reserve in Kenya
Source: PLoS One. 2020 Sep 18;15(9):e0239545. doi: 10.1371/journal.pone.0239545 (PMC7500588; doi:10.1371/journal.pone.0239545)
Supplement: S4 Table — (DOCX) [file pone.0239545.s007.docx]

**S4 Table: Propensity Scores and covariate balance**

|  |  | | **Mean** | | | **t-Value** | |  |
| --- | --- | --- | --- | --- | --- | --- | --- | --- |
| **Variable** | | **Sample** | | **Treated** | **Control** | |  | |
| Gender | | Unmatched | | 1.874 | 1.728 | | 1.232* | |
|  |  | Matched | | 1.769 | 1.778 | | -0.162 | |
| Age of respondent | | Unmatched | | 43.733 | 46.148 | | -1.688* | |
|  |  | Matched | | 44.648 | 46.148 | | -0.851 | |
| Number of assets owned | | Unmatched | | 1.350 | 1.315 | | 0.574 | |
|  |  | Matched | | 1.306 | 1.315 | | -0.135 | |
| Education level of respondent | | Unmatched | | 1.788 | 1.722 | | 0.594 | |
|  |  | Matched | | 1.704 | 1.722 | | -0.153 | |
| Ethnicity of respondent | | Unmatched | | 1.184 | 1.296 | | -2.301** | |
|  |  | Matched | | 1.269 | 1.296 | | -0.452 | |
| Occupation of respondent | | Unmatched | | 2.700 | 2.500 | | 1.226 | |
|  |  | Matched | | 2.550 | 2.500 | | 0.251 | |
| Number of income sources | | Unmatched | | 1.539 | 1.370 | | 2.096** | |
|  |  | Matched | | 1.350 | 1.370 | | 0.013 | |
| Household size | | Unmatched | | 4.088 | 3.889 | | 0.788 | |
|  |  | Matched | | 3.620 | 3.889 | | -0.945 | |
| Benefits from elephant conservation | | Unmatched | | 1.691 | 1.611 | | 1.341** | |
|  |  | Matched | | 1.574 | 1.611 | | -0.552 | |
| Propensity Scores | | Unmatched | | 0.661 | 0.564 | | 4.682*** | |
|  |  | Matched | | 0.564 | 0.564 | | -0.012 | |

Asterisks *, ** and *** indicate statistical significance at the 10% and 5% and 1% levels respectively.
