# Supplementary material for: Assessing impacts of human-elephant conflict on human wellbeing: An empirical analysis of communities living with elephants around Maasai Mara National Reserve in Kenya
Source: PLoS One. 2020 Sep 18;15(9):e0239545. doi: 10.1371/journal.pone.0239545 (PMC7500588; doi:10.1371/journal.pone.0239545)
Supplement: S5 Table — (DOCX) [file pone.0239545.s008.docx]

**S5 Table: Generalised linear model results for factors affecting wellbeing** **for matched samples**

| **Factors** | | **SWI** | **WI** | **ASI** | **FSI** | **SSI** | **EI** | **SI** | **NSI** | |
| --- | --- | --- | --- | --- | --- | --- | --- | --- | --- | --- |
| **(Intercept)** | | **59.202***** | **27.207***** | **63.39***** | **63.861***** | **45.535***** | **39.887***** | **37.424***** | **55.662***** | |
| **Gender (Female)** | | 0.076 | -0.714 | -0.104 | -2.324 | -3.141 | 2.326 | -0.018 | 0.016 | |
| **Education (None)** | | -9.99 | **11.275*** | -3.805 | 4.803 | 3.542 | 0.386 | 0.186 | -3.526 | |
| **Education (Primary)** | | -0.853 | 11.644 | -2.376 | 9.114 | 2.81 | N/A | -2.361 | 4.05 | |
| **Education (Secondary)** | | -2.605 | 8.732 | -8.767 | 9.408 | 7.653 | N/A | -5.365 | -9.881 | |
| **Employment (Yes)** | | -3.295 | 3.846 | -1.084 | -2.184 | -4.164 | N/A | 1.046 | -1.93 | |
| **Benefits (Yes)** | | -4.701 | 0.622 | 3.012 | 1.177 | -1.147 | 1.331 | **5.444*** | 1.491 | |
| **HEC (No)** | | 4.898 | **9.94***** | **-8.752***** | -5.013 | **6.543**** | **5.772**** | **10.057***** | 0.282 | |
| **Age** | | -0.084 | **0.218**** | -0.143 | **-0.313**** | 0.12 | -0.16 | 0.105 | -0.033 | |
| **Household Size** | | 0.611 | -0.135 | 0.699 | **-1.637**** | **-1.522**** | -0.018 | -0.424 | -0.592 | |
| **(Scale)** | | 485.846^b^ | 262.365^b^ | 409.841^b^ | 496.588^b^ | 351.043^b^ | 366.994^b^ | 505.859^b^ | 540.930^b^ | |
| Asterisks *, ** and *** indicate statistical significance at the 10% and 5% and 1% levels respectively. | | | | | | | | |  |  |
| ^b^: Maximum likelihood estimate  N/A: Education is one of the components of Education Indicator (EI). | | | | | | | | |  |  |
| SWI=Subjective Wellbeing Index; WI=Wealth Index; ASI=Access to Services Index; FSI=Food Security Index; SSI=Satisfaction with Services Index; EI=Education Index; SI=Social Index; NSI=Natural Sphere Index | | | | | | | | |  |  |
