# Supplementary material for: Assessing impacts of human-elephant conflict on human wellbeing: An empirical analysis of communities living with elephants around Maasai Mara National Reserve in Kenya
Source: PLoS One. 2020 Sep 18;15(9):e0239545. doi: 10.1371/journal.pone.0239545 (PMC7500588; doi:10.1371/journal.pone.0239545)
Supplement: S6 Table — (DOCX) [file pone.0239545.s009.docx]

**S6 Table: Generalised linear Model results for HEC and wellbeing for unmatched samples (N=367)**

| Parameter Estimates | | | | | | | | | | | |
| --- | --- | --- | --- | --- | --- | --- | --- | --- | --- | --- | --- |
| **Parameter** | **B** | **Std. Error** | **95% Wald Confidence Interval** | | | | **Hypothesis Test** | | | | |
|  |  |  | **Lower** | | **Upper** | | **Wald Chi-Square** | | | **df** | **Sig.** |
| (Intercept) | .928 | .1549 | .624 | 1.231 | | 35.858 | | 1 | | | **.000** |
| Subjective wellbeing | -.004 | .0011 | -.006 | -.002 | | 12.953 | | 1 | | | **.000** |
| Wealth Index | .002 | .0013 | -.001 | .004 | | 1.756 | | 1 | | | .185 |
| Access to services | .001 | .0011 | -.001 | .003 | | .967 | | 1 | | | .325 |
| Food security | .000 | .0014 | -.003 | .002 | | .121 | | 1 | | | .728 |
| Satisfaction with services | -.002 | .0014 | -.004 | .001 | | 1.633 | | 1 | | | .201 |
| Education | .000 | .0011 | -.002 | .002 | | .069 | | 1 | | | .793 |
| Social interaction | -.005 | .0011 | -.007 | -.003 | | 18.702 | | 1 | | | **.000** |
| Natural environment | .001 | .0012 | -.001 | .004 | | 1.074 | | 1 | | | .300 |
| (Scale) | .218 | .0161 | .188 | .252 | |  | | |  | |  |
