# Supplementary material for: Assessing impacts of human-elephant conflict on human wellbeing: An empirical analysis of communities living with elephants around Maasai Mara National Reserve in Kenya
Source: PLoS One. 2020 Sep 18;15(9):e0239545. doi: 10.1371/journal.pone.0239545 (PMC7500588; doi:10.1371/journal.pone.0239545)
Supplement: S7 Table — (DOCX) [file pone.0239545.s010.docx]

**S7 Table: Generalised linear model results for HEC and wellbeing for matched samples (N=216)**

| Parameter Estimates | | | | | | | | | | | | | |
| --- | --- | --- | --- | --- | --- | --- | --- | --- | --- | --- | --- | --- | --- |
| **Parameter** | **B** | | **Std. Error** | | **95% Wald Confidence Interval** | | | | **Hypothesis Test** | | | | |
|  |  |  |  |  | **Lower** | **Upper** | | | **Wald Chi-Square** | | **df** | **Sig.** | |
| (Intercept) | | 1.262 | .1939 | .882 | | | 1.642 | 42.372 | | 1 | | | **.000** |
| Subjective wellbeing | | -.002 | .0013 | -.004 | | | .001 | 1.745 | | 1 | | | .186 |
| Wealth Index | | -.008 | .0017 | -.012 | | | -.005 | 23.330 | | 1 | | | **.000** |
| Access to services | | .005 | .0015 | .002 | | | .008 | 10.369 | | 1 | | | **.001** |
| Food security | | .002 | .0013 | .000 | | | .005 | 3.013 | | 1 | | | .083 |
| Satisfaction with services | | -.004 | .0016 | -.007 | | | -.001 | 6.172 | | 1 | | | **.013** |
| Education | | -.004 | .0016 | -.007 | | | -.001 | 6.289 | | 1 | | | **.012** |
| Social interaction | | -.005 | .0013 | -.007 | | | -.002 | 12.509 | | 1 | | | **.000** |
| Natural environment | | .000 | .0014 | -.003 | | | .002 | .126 | | 1 | | | .723 |
| (Scale) | .197 | |  | |  |  | | |  | |  |  | |
