# Supplementary material for: Assessing impacts of human-elephant conflict on human wellbeing: An empirical analysis of communities living with elephants around Maasai Mara National Reserve in Kenya
Source: PLoS One. 2020 Sep 18;15(9):e0239545. doi: 10.1371/journal.pone.0239545 (PMC7500588; doi:10.1371/journal.pone.0239545)
Supplement: S2 Appendix — (DOCX) [file pone.0239545.s012.docx]

**Appendix 2: Wellbeing indicators development workflow**

Following is an outline of the wellbeing indicator development workflow and the key results of the process. Refer to Nyumba (2018:143-147) for detailed account of the process.


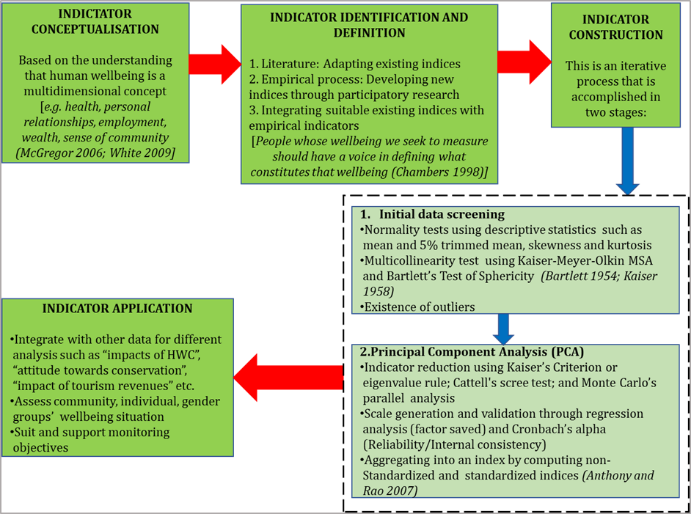


Wellbeing indicators development workflow

| **Descriptive statistics of the wellbeing variables** | | | | | |
| --- | --- | --- | --- | --- | --- |
|  | **Mean** | **5% Trimmed Mean** | **Range** | **Skewness** | **Kurtosis** |
| What is the highest education level category? | 2.02 | 1.97 | 3 | 0.570 | -0.666 |
| What is the highest education in the household? | 2.45 | 2.44 | 3 | 0.077 | -0.924 |
| How many assets do you have? | 2.63 | 2.68 | 2 | -1.052 | 0.037 |
| Does the household own a motorbike? | 1.69 | 1.71 | 1 | -0.808 | -1.354 |
| Quality and quantity of government support programmes in the last 12 months | 1.54 | 1.53 | 2 | 0.237 | -1.102 |
| Quality and quantity of Non-government support programmes in the last 12 months | 1.99 | 1.99 | 2 | -0.009 | 0.587 |
| Are the household’s sources of income regular? | 1.66 | 1.68 | 1 | -0.675 | -1.552 |
| Is your household prosperous? | 2.10 | 2.10 | 2 | 0.530 | 1.968 |
| Have there been any shortages of food for more than one month during the past 12 months? | 1.52 | 1.52 | 1 | -0.082 | -2.004 |
| Is the drinking water that you have access to clean? | 2.01 | 2.01 | 2 | -0.005 | -0.348 |
| What is the quality of water like in nearest river? | 1.84 | 1.82 | 2 | 0.318 | -1.729 |
| How are feelings of mutual trust among community members in the community? | 2.01 | 2.01 | 2 | 0.064 | 4.414 |
| Do conflicts arise between people or families in the community? | 2.13 | 2.12 | 2 | 0.857 | 1.882 |
| Do members of your household always receive modern medical treatment? | 1.74 | 1.73 | 3 | 0.271 | 2.245 |
| How much of the natural environment (e.g. forest, grassland, river) around your village is damaged? | 2.37 | 2.41 | 2 | -0.671 | -0.768 |
| How difficult is it to get to the nearest healthcare facility? | 1.81 | 1.80 | 2 | -0.355 | 0.261 |
| Are you satisfied with the quality of healthcare your family gets | 1.96 | 1.96 | 2 | -0.503 | 4.618 |
| Do you consider your household to be poor? | 2.14 | 2.15 | 2 | 0.343 | 0.757 |
| How difficult is it to get to the nearest primary school? | 1.65 | 1.66 | 2 | -0.495 | -1.393 |
| Are you satisfied with the quality of education that your family gets | 1.91 | 1.94 | 3 | -1.811 | 7.168 |
| How difficult is it to reach the nearest market? | 1.76 | 1.78 | 2 | -0.943 | -0.337 |
| How do you feel about your own health? | 2.24 | 2.21 | 4 | 0.761 | 0.843 |
| How do you feel about your shelter? | 2.46 | 2.43 | 4 | 0.624 | -0.175 |
| How do you feel about your achieving in life? | 2.45 | 2.41 | 4 | 0.529 | -0.385 |
| How do you feel about your personal relationships? | 2.43 | 2.40 | 4 | 0.620 | -0.144 |
| How do you feel about your personal future security? | 2.53 | 2.49 | 4 | 0.386 | -0.832 |
| How do you feel about the availability of water for your livestock? | 2.59 | 2.54 | 4 | 0.400 | -0.740 |
| How do you feel about your personal safety? | 2.58 | 2.53 | 4 | 0.567 | -0.426 |

| **Indicator loadings and communalities based on a PCA with Varimax rotation for 28 items (N =367)** | | | | | | | | | | | |
| --- | --- | --- | --- | --- | --- | --- | --- | --- | --- | --- | --- |
| **Items** | **Indicator** | | | | | | | | | | |
|  | **1** | **2** | **3** | **4** | **5** | **6** | **7** | **8** | **9** | **10** |  |
| How do you feel about your personal future security? | 0.740 |  |  |  |  |  |  |  |  |  |  |
| How do you feel about your personal relationships? | 0.726 |  |  |  |  |  |  |  |  |  |  |
| How do you feel about your achieving in life? | 0.725 |  |  |  |  |  |  |  |  |  |  |
| How do you feel about your personal safety? | 0.669 |  |  |  |  |  |  |  |  |  |  |
| How do you feel about your shelter? | 0.609 |  |  |  |  |  |  |  |  |  |  |
| How do you feel about the availability of water for your livestock? | 0.558 |  |  |  |  |  |  |  |  |  |  |
| Are the household’s sources of income regular? |  | 0.849 |  |  |  |  |  |  |  |  |  |
| How many assets do you have? |  | 0.821 |  |  |  |  |  |  |  |  |  |
| Does the household own a motorbike? |  | 0.810 |  |  |  |  |  |  |  |  |  |
| How difficult is it to get to the nearest primary school? |  |  | 0.848 |  |  |  |  |  |  |  |  |
| How difficult is it to reach the nearest market? |  |  | 0.816 |  |  |  |  |  |  |  |  |
| How difficult is it to get to the nearest healthcare facility |  |  | 0.746 |  |  |  |  |  |  |  |  |
| Have there been any shortages of food for more than one month during the past 12 months? |  |  |  | 0.782 |  |  |  |  |  |  |  |
| Is your household prosperous? |  |  |  | 0.699 |  |  |  |  |  |  |  |
| Do you consider your household to be poor? |  |  |  | 0.675 |  |  |  |  |  |  |  |
| Are you satisfied with the quality of healthcare your family gets |  |  |  |  | 0.822 |  |  |  |  |  |  |
| Are you satisfied with the quality of education your family gets |  |  |  |  | 0.750 |  |  |  |  |  |  |
| What is the highest education level in the household? |  |  |  |  |  | 0.903 |  |  |  |  |  |
| What is the highest education level of the respondent? |  |  |  |  |  | 0.894 |  |  |  |  |  |
| How are feelings of mutual trust among community members in the community? |  |  |  |  |  |  | 0.829 |  |  |  |  |
| Do conflicts arise between people or families in the community? |  |  |  |  |  |  | 0.823 |  |  |  |  |
| How much of the natural environment around your village is damaged? |  |  |  |  |  |  |  | 0.845 |  |  |  |
| What is the quality of water like in nearest river? |  |  |  |  |  |  |  | 0.795 |  |  |  |
| Quality and quantity of Non-government support programmes in the last 12 months |  |  |  |  |  |  |  |  | 0.712 |  |  |
| Quality and quantity of government support programmes in the last 12 months |  |  |  |  |  |  |  |  | 0.635 |  |  |
| Do members of your household always receive modern medical treatment? |  |  |  |  |  |  |  |  | 0.520 |  |  |
| How do you feel about your own health? |  |  |  |  |  |  |  |  |  | 0.676 |  |
| Is the drinking water that you have access to clean? |  |  |  |  |  |  |  |  |  | 0.578 |  |
| Extraction Method: Principal Component Analysis; Rotation converged in 8 iterations; Factor loadings < 0.3 are suppressed | | | | | | | | | | | |

**Reliability and descriptive statistics of wellbeing dimensions (N =367)**

| **Index** | **Items** | ***M*** | ***SD*** | ***Alpha (α)*** |
| --- | --- | --- | --- | --- |
| Subjective Wellbeing | How do you feel about your personal future security | 2.53 | 1.12 | 0.759 |
|  | How do you feel about your personal relationships? | 2.43 | 0.98 |  |
|  | How do you feel about your achieving in life? | 2.45 | 1.02 |  |
|  | How do you feel about your personal safety? | 2.58 | 1.09 |  |
|  | How do you feel about your shelter? | 2.46 | 0.95 |  |
|  | How do you feel about the availability of water for your livestock and crops? | 2.59 | 1.16 |  |
| Wealth | Are sources of household income regular? | 1.79 | 0.41 | 0.818 |
|  | How many assets do you have? | 2.63 | 0.53 |  |
|  | Does the household own a motorbike? | 1.69 | 0.46 |  |
| Access to Services | How difficult is it to get to the nearest primary school? | 1.65 | 0.49 | 0.800 |
|  | How difficult is it to get to the nearest market? | 1.76 | 0.45 |  |
|  | How difficult is it to get to the nearest healthcare facility? | 1.81 | 0.50 |  |
| Food Security | Have there been any shortages of food for more than one month during the past 12 months? | 1.52 | 0.50 | 0.675 |
|  | Is your household prosperous? | 2.10 | 0.43 |  |
|  | Do you consider your household to be poor? | 2.14 | 0.48 |  |
| Satisfaction with Services | How satisfied are you with the healthcare your family gets? | 1.96 | 0.36 | 0.660 |
|  | How satisfied are you with the education they get? | 1.91 | 0.41 |  |
| Education | What is the highest education level of respondents? | 2.02 | 0.95 | 0.809 |
|  | What is the highest education level in the household? | 2.45 | 0.96 |  |
| Social | How are feelings of mutual trust among community members in the community? | 2.01 | 0.37 | 0.696 |
|  | Do conflicts arise between people or families in the community? | 2.13 | 0.41 |  |
| Natural Sphere | How much of the natural environment around your village is damaged? | 2.25 | 0.67 | 0.617 |
|  | What is the quality of water like in nearest river? | 2.93 | 0.49 |  |
| Support Programmes | Quality and quantity of Non-government support programmes in the last 12 months | 1.99 | 0.53 | 0.342 |
|  | Quality and quantity of government support programmes in the last 12 months | 1.89 | 0.37 |  |
|  | Do members of your household always receive modern medical treatment? | 1.74 | 0.53 |  |
| Health Index | How do you feel about your own health? | 2.24 | 0.77 | 0.070 |
|  | Is the drinking water that you have access to clean? | 2.15 | 0.46 |  |
