# Supplementary material for: Assessing impacts of human-elephant conflict on human wellbeing: An empirical analysis of communities living with elephants around Maasai Mara National Reserve in Kenya
Source: PLoS One. 2020 Sep 18;15(9):e0239545. doi: 10.1371/journal.pone.0239545 (PMC7500588; doi:10.1371/journal.pone.0239545)
Supplement: S3 Appendix — (DOCX) [file pone.0239545.s013.docx]

# Appendix 2: Testing Assumptions of Linear Regression

1. **Testing for autocorrelation**

We tested for autocorrelation using Durbin-Watson test in SPSS.  The Durbin-Watson tests produces a test statistic that ranges from 0 to 4.  Values close to 2 (the middle of the range) suggest less autocorrelation, and values closer to 0 or 4 indicate greater positive or negative autocorrelation respectively.

| **Indicator** | **R** | **R Square** | **Adjusted R Square** | **Std. Error of the Estimate** | **Durbin-Watson** |
| --- | --- | --- | --- | --- | --- |
| Subjective Wellbeing | .208^a^ | .043 | .011 | 22.56556 | 1.951 |
| Wealth Indicator | .356^a^ | .126 | .097 | 16.56205 | 2.108 |
| Access to Services | .252^a^ | .064 | .032 | 20.73370 | 2.021 |
| Food Security | .284^a^ | .080 | .050 | 22.79723 | 1.743 |
| Satisfaction with Services | .278^a^ | .077 | .046 | 19.16466 | 0.188 |
| Education | .212^a^ | .045 | .013 | 19.42940 | 1.885 |
| Social Interactions | .261^a^ | .068 | .037 | 22.95056 | 1.788 |
| Natural Environment | .076^a^ | .006 | -.028 | 24.09497 | 1.916 |

^a.^ **Predictors:** (Constant), Did you have problems with elephants in the last 12months? Education level of respondent, Age of respondent, Benefits from elephant conservation, Household size, Gender, Employed?

1. **Testing for multicollinearity**

We tested for multicollinearity using the Variance of Inflation (VIF). All the variables in each model for wellbeing indicators returned VIFs of less than 10.

| **Subjective Wellbeing** | | | | | | | | |
| --- | --- | --- | --- | --- | --- | --- | --- | --- |
| Model | | Unstandardized Coefficients | | Standardized Coefficients | t | Sig. | Collinearity Statistics | |
|  |  | B | Std. Error | Beta |  |  | Tolerance | VIF |
| 1 | (Constant) | 38.293 | 12.755 |  | 3.002 | .003 |  |  |
|  | Gender | -.190 | 3.784 | -.004 | -.050 | .960 | .939 | 1.065 |
|  | Age of respondent | -.082 | .122 | -.047 | -.676 | .500 | .955 | 1.047 |
|  | Education level of respondent | 3.864 | 1.784 | .152 | 2.166 | .031 | .940 | 1.063 |
|  | Employed? | 1.802 | 3.415 | .038 | .528 | .598 | .881 | 1.135 |
|  | Household size | .611 | .750 | .056 | .815 | .416 | .967 | 1.034 |
|  | Benefits from elephant conservation | 4.440 | 3.222 | .096 | 1.378 | .170 | .941 | 1.063 |
|  | Did you have problems with elephants in the last 12months? | -4.685 | 3.091 | -.103 | -1.516 | .131 | .987 | 1.013 |

| **Wealth Indicator** | | | | | | | | |
| --- | --- | --- | --- | --- | --- | --- | --- | --- |
| Model | | Unstandardized Coefficients | | Standardized Coefficients | t | Sig. | Collinearity Statistics | |
|  |  | B | Std. Error | Beta |  |  | Tolerance | VIF |
| 1 | (Constant) | 60.563 | 9.362 |  | 6.469 | .000 |  |  |
|  | Gender | .361 | 2.777 | .009 | .130 | .897 | .939 | 1.065 |
|  | Age of respondent | .212 | .089 | .158 | 2.375 | .018 | .955 | 1.047 |
|  | Education level of respondent | -1.842 | 1.309 | -.094 | -1.407 | .161 | .940 | 1.063 |
|  | Employed? | -4.486 | 2.507 | -.124 | -1.790 | .075 | .881 | 1.135 |
|  | Household size | -.163 | .550 | -.020 | -.296 | .767 | .967 | 1.034 |
|  | Benefits from elephant conservation | -.784 | 2.365 | -.022 | -.332 | .741 | .941 | 1.063 |
|  | Did you have problems with elephants in the last 12months? | -9.767 | 2.269 | -.281 | -4.305 | .000 | .987 | 1.013 |

| **Access to Services** | | | | | | | | |
| --- | --- | --- | --- | --- | --- | --- | --- | --- |
| Model | | Unstandardized Coefficients | | Standardized Coefficients | t | Sig. | Collinearity Statistics | |
|  |  | B | Std. Error | Beta |  |  | Tolerance | VIF |
| 1 | (Constant) | 55.849 | 11.720 |  | 4.765 | .000 |  |  |
|  | Gender | .559 | 3.477 | .011 | .161 | .872 | .939 | 1.065 |
|  | Age of respondent | -.130 | .112 | -.080 | -1.166 | .245 | .955 | 1.047 |
|  | Education level of respondent | -1.310 | 1.639 | -.055 | -.799 | .425 | .940 | 1.063 |
|  | Employed? | .333 | 3.138 | .008 | .106 | .916 | .881 | 1.135 |
|  | Household size | .747 | .689 | .074 | 1.084 | .280 | .967 | 1.034 |
|  | Benefits from elephant conservation | -3.060 | 2.960 | -.072 | -1.033 | .303 | .941 | 1.063 |
|  | Did you have problems with elephants in the last 12months? | 8.722 | 2.840 | .207 | 3.071 | .002 | .987 | 1.013 |

**Food Security**

| Model | | Unstandardized Coefficients | | Standardized Coefficients | t | Sig. | Collinearity Statistics | |
| --- | --- | --- | --- | --- | --- | --- | --- | --- |
|  |  | B | Std. Error | Beta |  |  | Tolerance | VIF |
| 1 | (Constant) | 59.612 | 12.886 |  | 4.626 | .000 |  |  |
|  | Gender | 1.777 | 3.823 | .032 | .465 | .643 | .939 | 1.065 |
|  | Age of respondent | -.323 | .123 | -.179 | -2.628 | .009 | .955 | 1.047 |
|  | Education level of respondent | 1.380 | 1.802 | .053 | .766 | .445 | .940 | 1.063 |
|  | Employed? | 1.331 | 3.450 | .027 | .386 | .700 | .881 | 1.135 |
|  | Household size | -1.682 | .758 | -.150 | -2.220 | .027 | .967 | 1.034 |
|  | Benefits from elephant conservation | -1.405 | 3.255 | -.030 | -.432 | .666 | .941 | 1.063 |
|  | Did you have problems with elephants in the last 12months? | 5.264 | 3.123 | .113 | 1.686 | .093 | .987 | 1.013 |

| **Satisfaction with Services** | | | | | | | | |
| --- | --- | --- | --- | --- | --- | --- | --- | --- |
| Model | | Unstandardized Coefficients | | Standardized Coefficients | t | Sig. | Collinearity Statistics | |
|  |  | B | Std. Error | Beta |  |  | Tolerance | VIF |
| 1 | (Constant) | 37.987 | 10.833 |  | 3.507 | .001 |  |  |
|  | Gender | 2.741 | 3.214 | .059 | .853 | .395 | .939 | 1.065 |
|  | Age of respondent | .109 | .103 | .072 | 1.056 | .292 | .955 | 1.047 |
|  | Education level of respondent | 1.073 | 1.515 | .049 | .709 | .479 | .940 | 1.063 |
|  | Employed? | 4.653 | 2.901 | .114 | 1.604 | .110 | .881 | 1.135 |
|  | Household size | -1.563 | .637 | -.166 | -2.454 | .015 | .967 | 1.034 |
|  | Benefits from elephant conservation | 1.161 | 2.736 | .029 | .424 | .672 | .941 | 1.063 |
|  | Did you have problems with elephants in the last 12months? | -6.495 | 2.625 | -.166 | -2.474 | .014 | .987 | 1.013 |

| **Education** | | | | | | | | |
| --- | --- | --- | --- | --- | --- | --- | --- | --- |
| Model | | Unstandardized Coefficients | | Standardized Coefficients | t | Sig. | Collinearity Statistics | |
|  |  | B | Std. Error | Beta |  |  | Tolerance | VIF |
| 1 | (Constant) | 60.498 | 10.982 |  | 5.509 | .000 |  |  |
|  | Gender | -2.657 | 3.258 | -.057 | -.815 | .416 | .939 | 1.065 |
|  | Age of respondent | -.161 | .105 | -.107 | -1.539 | .125 | .955 | 1.047 |
|  | Education level of respondent | -2.165 | 1.536 | -.099 | -1.410 | .160 | .940 | 1.063 |
|  | Employed? | -1.187 | 2.941 | -.029 | -.404 | .687 | .881 | 1.135 |
|  | Household size | -.088 | .646 | -.009 | -.136 | .892 | .967 | 1.034 |
|  | Benefits from elephant conservation | -1.825 | 2.774 | -.046 | -.658 | .511 | .941 | 1.063 |
|  | Did you have problems with elephants in the last 12months? | -5.803 | 2.662 | -.149 | -2.180 | .030 | .987 | 1.013 |

| **Social Interactions** | | | | | | | | |
| --- | --- | --- | --- | --- | --- | --- | --- | --- |
| Model | | Unstandardized Coefficients | | Standardized Coefficients | t | Sig. | Collinearity Statistics | |
|  |  | B | Std. Error | Beta |  |  | Tolerance | VIF |
| 1 | (Constant) | 60.969 | 12.973 |  | 4.700 | .000 |  |  |
|  | Gender | .379 | 3.849 | .007 | .098 | .922 | .939 | 1.065 |
|  | Age of respondent | .113 | .124 | .063 | .916 | .361 | .955 | 1.047 |
|  | Education level of respondent | -2.054 | 1.814 | -.078 | -1.133 | .259 | .940 | 1.063 |
|  | Employed? | -.890 | 3.474 | -.018 | -.256 | .798 | .881 | 1.135 |
|  | Household size | -.391 | .763 | -.035 | -.512 | .609 | .967 | 1.034 |
|  | Benefits from elephant conservation | -5.359 | 3.277 | -.113 | -1.635 | .103 | .941 | 1.063 |
|  | Did you have problems with elephants in the last 12months? | -10.173 | 3.144 | -.218 | -3.236 | .001 | .987 | 1.013 |

| **Natural Environment** | | | | | | | | |
| --- | --- | --- | --- | --- | --- | --- | --- | --- |
| Model | | Unstandardized Coefficients | | Standardized Coefficients | t | Sig. | Collinearity Statistics | |
|  |  | B | Std. Error | Beta |  |  | Tolerance | VIF |
| 1 | (Constant) | 57.303 | 13.620 |  | 4.207 | .000 |  |  |
|  | Gender | .439 | 4.040 | .008 | .109 | .914 | .939 | 1.065 |
|  | Age of respondent | -.014 | .130 | -.008 | -.112 | .911 | .955 | 1.047 |
|  | Education level of respondent | -1.515 | 1.904 | -.057 | -.795 | .427 | .940 | 1.063 |
|  | Employed? | -.612 | 3.647 | -.012 | -.168 | .867 | .881 | 1.135 |
|  | Household size | -.531 | .801 | -.047 | -.664 | .508 | .967 | 1.034 |
|  | Benefits from elephant conservation | -1.829 | 3.440 | -.038 | -.532 | .595 | .941 | 1.063 |
|  | Did you have problems with elephants in the last 12months? | -.093 | 3.301 | -.002 | -.028 | .977 | .987 | 1.013 |

1. **Testing for heteroskedasticity**

We used Breusch-Pagan test in addition to visual verification of P-P Plots to test for heteroscedasticity in our data before analysis. The BP test postulates that if the p-value associated with a heteroscedasticity test falls below a certain threshold (0.05 for example), we would conclude that the data is significantly heteroscedastic. For this study, we set the P-Value at 0.005. In our data, two parameters, Satisfaction with Services Index (P<0.005) and Social Index (p<0.005), had *p*-values suggesting they heteroscedastic.

**Results of the heteroskedasticity tests using Breusch-Pagan test in SPSS**

| Indicator Model | | Sum of Squares | df | Mean Square | F | Sig. |
| --- | --- | --- | --- | --- | --- | --- |
| SW | Regression | 1135319.469 | 7 | 162188.496 | .392 | .906^b^ |
|  | Residual | 85980070.456 | 208 | 413365.723 |  |  |
|  | Total | 87115389.925 | 215 |  |  |  |
| WI | Regression | 559795.916 | 7 | 79970.845 | .749 | .631^b^ |
|  | Residual | 22217177.674 | 208 | 106813.354 |  |  |
|  | Total | 22776973.590 | 215 |  |  |  |
| ASI | Regression | 487471.301 | 7 | 69638.757 | .220 | .980^b^ |
|  | Residual | 65901885.064 | 208 | 316835.986 |  |  |
|  | Total | 66389356.365 | 215 |  |  |  |
| FSI | Regression | 2396699.836 | 7 | 342385.691 | .964 | .459^b^ |
|  | Residual | 73891642.210 | 208 | 355248.280 |  |  |
|  | Total | 76288342.046 | 215 |  |  |  |
| SSI | Regression | 5225729.950 | 7 | 746532.850 | 3.234 | .003^b^ |
|  | Residual | 48021316.936 | 208 | 230871.716 |  |  |
|  | Total | 53247046.886 | 215 |  |  |  |
| EI | Regression | 2234439.564 | 7 | 319205.652 | 1.520 | .162^b^ |
|  | Residual | 43668529.669 | 208 | 209944.854 |  |  |
|  | Total | 45902969.233 | 215 |  |  |  |
| SI | Regression | 13350516.578 | 7 | 1907216.654 | 4.975 | .000^b^ |
|  | Residual | 79746474.200 | 208 | 383396.511 |  |  |
|  | Total | 93096990.778 | 215 |  |  |  |
| NSI | Regression | 1173424.057 | 7 | 167632.008 | .477 | .851^b^ |
|  | Residual | 73103030.803 | 208 | 351456.879 |  |  |
|  | Total | 74276454.860 | 215 |  |  |  |

SWI=Subjective Wellbeing Index; WI=Wealth Index; ASI=Access to Services Index; FSI=Food Security Index; SSI=Satisfaction with Services Index; EI=Education Index; SI=Social Index; NSI=Natural Sphere Index
